# Interaction of Red Cabbage Extract with Exogenous Antioxidants

**DOI:** 10.3390/ijms262211011

**Published:** 2025-11-14

**Authors:** Kacper Kut, Oskar Sitarz, Ireneusz Kapusta, Grzegorz Bartosz, Izabela Sadowska-Bartosz

**Affiliations:** 1 Laboratory of Analytical Biochemistry, Institute of Food Technology and Nutrition, Faculty of Technology and Life Sciences, University of Rzeszow, 35-601 Rzeszow, Poland; kkut@ur.edu.pl (K.K.); sitarz11@o2.pl (O.S.); gbartosz@ur.edu.pl (G.B.); 2 Doctoral School, University of Rzeszow, 35-310 Rzeszow, Poland; 3 Department of General Food Technology and Human Nutrition, Faculty of Technology and Life Sciences, University of Rzeszow, 35-601 Rzeszow, Poland; ikapusta@ur.edu.pl

**Keywords:** additivity, antagonism, antioxidant interaction, ascorbic acid, gallic acid, glutathione, red cabbage, TEMPOL, Trolox, synergy

## Abstract

Interactions between antioxidants are of interest, mainly for understanding their action in complex biological and food systems. This study aimed to evaluate interactions between the anthocyanin-rich aqueous red cabbage extract and several natural (ascorbic acid, gallic acid, and glutathione) and synthetic (Trolox and TEMPOL) antioxidants as a function of reaction time, concentration, and extract/antioxidant ratio in the ABTS^•^ decolorization and FRAP antioxidant activity assays. The measured interaction type showed dependence on assay type, reaction time, and the extract/antioxidant ratio, but no general dependence on the concentrations of the extract and the antioxidants over a 6-fold concentration range. In the ABTS^•^ decolorization assay, the interactions between the red cabbage extract and exogenous antioxidants were additive (Trolox), weakly antagonistic (ascorbic acid, gallic acid, and glutathione), and definitely antagonistic for TEMPOL. In the FRAP assay, the interactions were additive (ascorbic acid and Trolox), weakly antagonistic (gallic acid and TEMPOL), and definitely synergistic for glutathione. These results suggest the need for a series of assays covering a range of conditions to demonstrate a deviation from additivity in the interactions between antioxidants. The synergy of the interaction of glutathione with the extract in the FRAP assay indicates the possibility of a higher reactivity of this compound in Fe^3+^ reduction in complex systems than in an assay of the pure compound.

## 1. Introduction

According to the classical chemical definition, antioxidant is a “substance that, when present at low concentrations compared with that of an oxidizable substrate, significantly delays or inhibits oxidation of that substrate” [1]. This definition, appropriate for chemical reactions, is not sufficient for complex biological systems. The definition proposed by the Panel on Dietary Antioxidants and Related Compounds of the Food and Nutrition Board is that “a dietary antioxidant is a substance in food that significantly decreases the adverse effects of reactive oxygen species (ROS), reactive nitrogen species, or both on normal physiological function in human” [2]. A simple definition of an antioxidant as “a molecule capable of inhibiting the oxidation of other molecules” [3] is perhaps the most suitable for in vitro reactions since antioxidants are not always present in small amounts in the system. Enormous knowledge has been accumulated on the properties and biological roles of antioxidants, as well as their usefulness in food preservation, disease prophylaxis, and therapy. The PubMed database returns about 814,000 results for the keyword “antioxidant,” while Google Scholar returns about 3.8 million (October 2025). Studies of individual antioxidants have enabled the understanding of their mechanisms of action at various levels. However, interactions between antioxidants are of interest, mainly for understanding their action in complex biological and food systems. In food systems, knowledge and understanding of such interactions may have a practical dimension, as they may allow for reducing the amount of exogenous antioxidants needed to prevent undesired oxidation reactions [4,5,6].

Interactions between antioxidants are studied at various levels. One of the basic fields of such studies covers the interaction between antioxidants in antioxidant activity assays. Addition of two or more antioxidants causes, in many cases, a response equal (within the limits of experimental error) to the sum of responses to individual antioxidants (additivity). However, in some cases, the effect is greater or smaller than the sum of the individual antioxidants’ impacts (synergy or antagonism, respectively). e.g., in the FRAP assay, reduction of Fe^3+^ by a mixture of 3.2 µM *p*-coumaric acid and 3.2 µM sinapic acid was found to correspond to 171.5% of that equal to the sum of reduction by 3.2 µM *p*-coumaric acid and 3.2 µM sinapic acid acting separately (synergy) [7]. In the same assay, the effect of a mixture of 2.94 µM gallic acid and 11.8 µM caffeic acid was 237.8% of the sum of the effects of the individual components acting separately (synergy) [8]. In the ABTS^•^ decolorization assay, a mixture of kaempferol and gallic acid showed activity equal to 70% of the sum of activities of these compounds assayed individually (antagonism). In comparison, the antioxidant activity of a mixture of catechin and dodecyl gallate was 111% of the sum of activities of individual compounds (synergy) [9]. In the same assay, a mixture of 0.7 µM quercetin and 10 µM glutathione yielded 89.5% of the decolorization expected from the summation of activities of individual compounds. In comparison, a mixture of 2.3 µM quercetin and 2.6 µM glutathione acted additively (97.1% of the sum of activities of individual components) [10]. In the DPPH^•^ decolorization assay, the effect of a mixture of 0.1 mM gallic acid and 0.1 mM procatechuic acid was equal to 141.5% of that predicted as the sum of activities of individual compounds (synergy). For a mixture of 0.1 mM gallic acid and 0.1 mM vanillic acid, an analogous value was 91.7% (antagonism) [11]. In the same assay, the effect of a mixture of quercetin (3.3 µM) and resveratrol at a 1:3 molar ratio was 89.1% of the impact expected for the sum of the individual components (antagonism). In contrast, for a mixture in a molar ratio of 3:1, the effect was 125.5% of the expected one (synergism) [12]. An extensive review of additive, synergic, and antagonistic interactions in binary systems has been published [13].

Antioxidant activity of mixtures of three or more individual antioxidants may also show synergy or antagonism, although a tendency towards additivity is often observed as the number of components increases. It was repeatedly found that the interaction effect may depend on component concentrations [7,10,14,15].

In this study, a different approach to examining antioxidant interactions was adopted. Instead of using single antioxidants, we investigated the interactions between an aqueous extract of red cabbage and exogenous antioxidants. The red cabbage extract is rich in anthocyanins (various derivatives of cyanidin) [16,17] but also contains other endogenous antioxidants. This system is closer to real systems in which antioxidant supplementation affects cells, organisms, or food products that already possess endogenous antioxidants.

We used three natural antioxidants (ascorbic acid, gallic acid, and glutathione) and two synthetic antioxidants, Trolox and TEMPOL. Ascorbic acid (vitamin C) is the main water-soluble antioxidant vitamin, essential for preventing connective tissue diseases and exerting many other beneficial health effects [18,19]. Gallic acid (3,4,5-trihydroxybenzoic acid) is one of the main phenolic acids found in plants, is of great importance in numerous industries, and exerts multiple health effects in humans [20,21]. Glutathione is the main intracellular antioxidant in animal cells and is also present in most other organisms [22,23]. Trolox is an analog of vitamin E, of appreciable water solubility, which, in combination with a considerable hydrophobicity, makes it a convenient and commonly used standard antioxidant [24,25]. TEMPOL is a stable nitroxide free radical broadly used as an antioxidant [26,27].

The specific objective of the study was to identify factors affecting the value of the interaction coefficient by examining the dependence of the interaction between extract antioxidants and exogenous antioxidants on the ratio of reagents, time of reaction, reaction rate ratio of the interacting antioxidants, and their concentration in two different assays of antioxidant activity.

## 2. Results

### 2.1. Chemical Composition of the Extract

The chemical composition of the extract, presented in Table 1, indicates that polyphenols, and among them anthocyanins, are the main components of the extract.

The composition of polyphenols in the extract is presented in Table 2. The anthocyanin pool is composed of various cyanidin derivatives, while other phenolics consist mainly of sinapoyl derivatives.

### 2.2. Interactions in the ABTS^•^ Decolorization Assay

**Figure 1:**
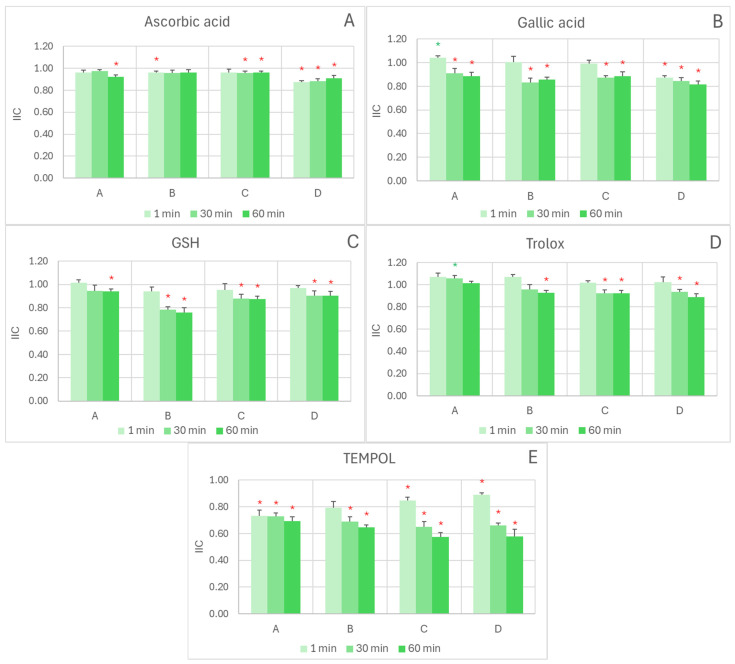
Effect of reagent ratio and reaction time on the integrated interaction coefficient (IIC) in the exogenous antioxidant/red cabbage extract systems measured by the ABTS^•^ decolorization assay. The concentrations of antioxidants introduced to the wells (1–6 µL) were as follows: (**A**)-A, 0.04 mM AC, 1.6 mM ascorbic acid; (**A**)-B, 0.08 mM AC, 1.2 mM ascorbic acid; (**A**)-C, 0.12 mM AC, 0.8 mM ascorbic acid; (**A**)-D; 0.12 mM AC, 0.4 mM ascorbic acid; (**B**)-A: 0.04 mM AC, 0.4 mM gallic acid; (**B**)-B, 0.08 mM AC, 0.3 mM gallic acid; (**B**)-C, 0.12 mM AC, 0.2 mM gallic acid; (**B**)-D, 0.16 mM AC, 0.1 mM gallic acid; (**C**)-A, 0.04 mM AC, 0.8 mM GSH; (**C**)-B, 0.08 mM AC, 0.6 mM GSH; (**C**)-C, 0.12 mM AC, 0.4 mM GSH; (**C**)-D, 0.16 mM AC, 0.2 mM GSH; (**D**)-A, 0.04 mM AC, 1.44 mM Trolox; (**D**)-B, 0.08 mM AC, 1,08 mM Trolox; (**D**)-C, 0.12 mM AC, 0.72 mM Trolox; (**D**)-D, 0.16 mM AC, 0.36 mM Trolox; (**E**)-A, 0.04 mM AC, 40 mM TEMPO; (**E**)-B, 0.08 mM AC, 30 mM TEMPOL; (**E**)-C, 0.12 mM AC, 20 mM TEMPOL; (**E**)-D, 0.16 mM AC, 10 mM TEMPOL. Reaction time: 1, 30, and 60 min. * *p* < 0.05 with respect to additive interaction (IIC = 1) (one-tailed Student “*t*” test). Green asterisks indicate synergic interaction, red asterisks denote antagonistic interaction; the lack of an asterisk indicates additive interaction. AC, anthocyanins.

In the ABTS^•^ decolorization assay, the interactions with exogenous antioxidants were mostly additive or antagonistic, as evidenced by the integrated interaction coefficient (IIC) ≤ 1. The type of interaction changed with reaction time and reagent ratio in some cases. In all cases, the short reaction time (1 min) favored additive (Figure 1A,C,D) or even synergic (Figure 1B) interactions, while longer reaction times (30 and 60 min) resulted in weakly antagonistic interactions (Figure 1A–D). Weakly synergic effects were found for the interaction of the extract with gallic acid at the lowest extract/gallic acid ratio (IIC of 1.04 ± 0.02) after a 1 min reaction (Figure 1B) and for the interaction of the extract with Trolox, also at the lowest extract/antioxidant ratio (IIC of 1.06 ± 0.02) after a 30 min reaction (Figure 1D). The interaction between the extract and TEMPOL was antagonistic under all conditions tested (Figure 1E).

To explain the observed dependencies, the ratio of the reaction rates of the extract and exogenous antioxidants was compared as a function of the extract/antioxidant ratio and reaction time. For ascorbic acid (Trolox), the ratio of the extract/antioxidant reaction rates increased with increasing reaction time, indicating that (some) components of the extract react with ABTS^•^ at slower rates than ascorbate and Trolox. For gallic acid and glutathione, this trend was observed only for lower extract/antioxidant ratios. For TEMPOL, the reaction rate decreased with increasing reaction times, which confirms the low reaction rate of TEMPOL with ABTS^•^ (Table S1). In most cases, the IIC values decreased with increasing reaction times. The correlation coefficients, calculated for all extract/antioxidant ratios, were positive for ascorbic acid and negative for gallic acid, glutathione, Trolox, and TEMPOL, and were statistically significant for gallic acid, Trolox, and TEMPOL (*p* values in bold, Table 3).

**Figure 2:**
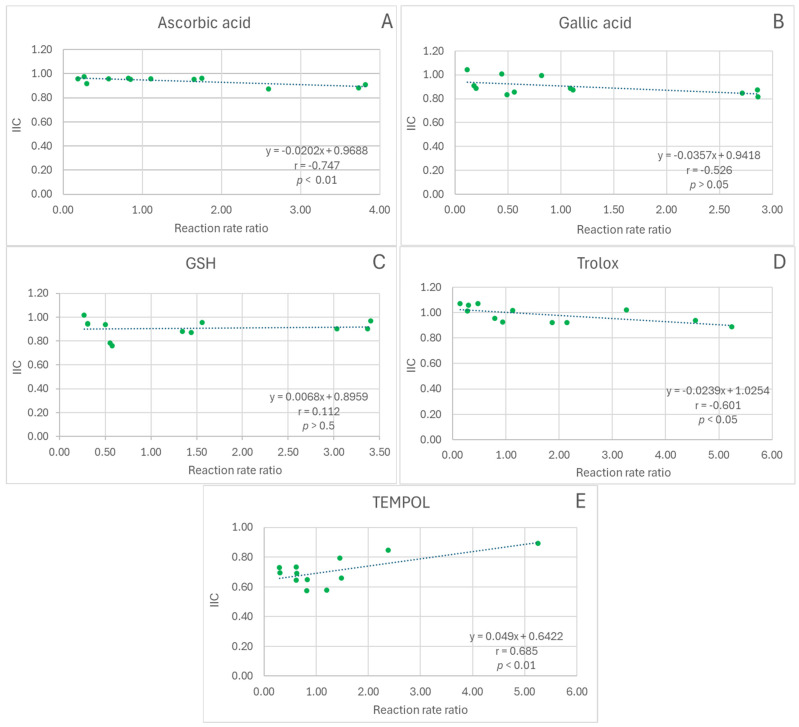
Dependence of IIC on the reaction rate ratio in the ABTS^•^ decolorization assay. (**A**), red cabbage extract + ascorbic acid; (**B**), red cabbage extract + gallic acid; (**C**), red cabbage extract + glutathione; (**D**), red cabbage extract + Trolox; (**E**), red cabbage extract + TEMPO; r, Pearson correlation coefficient; *p*, statistical significance of the correlation coefficient.

The dependence of the IIC on the extract/antioxidant reaction rate ratio was not significant for gallic acid and glutathione (Figure 2B,C), negative for ascorbic acid and Trolox (correlation coefficients statistically significant, *p* < 0.01 and *p* < 0.05, respectively; Figure 2A,D), and positive for TEMPOL (correlation coefficient statistically significant, *p* < 0.01, Figure 2E).

### 2.3. Interactions in the FRAP Assay

**Figure 3:**
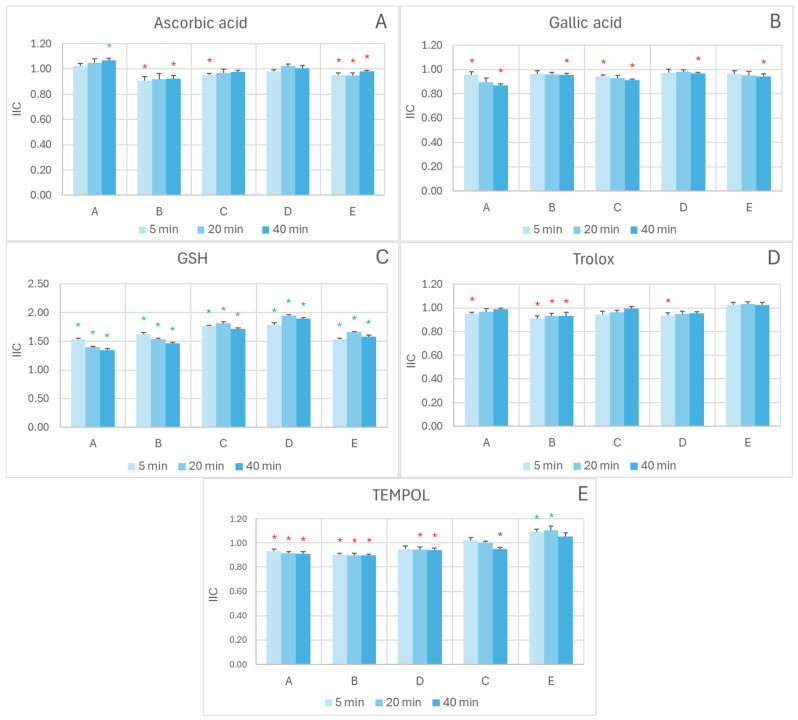
Effect of reagent ratio and reaction time on the IIC in the exogenous antioxidant/red cabbage/systems measured by the FRAP assay. The concentrations of antioxidants introduced to the wells (1–6 µL) were as follows: (**A**)-A, 0.1 mM AC, 1 mM ascorbic acid; (**A**)-B, 0.15 mM AC, 0.8 mM ascorbic acid (**A**)-C, 0.2 mM AC, 0.6 mM ascorbic acid; (**A**)-D, 0.3 mM AC, 0.4 mM ascorbic acid; (**A**)-E, 0.4 mM AC; 0.2 mM ascorbic acid; (**B**)-A, 0.1 mM AC, 1 mM gallic acid; (**B**)-B, 0.15 mM AC, 0.8 mM gallic acid; (**B**)-C, 0.2 mM AC, 0.6 mM gallic acid; (**B**)-D, 0.3 mM AC, 0.4 mM gallic acid; (**B**)-E, 0.4 mM AC; 0.2 mM gallic acid; (**C**)-A, 0.1 mM AC, 5 mM GSH; (**C**)-B, 0.15 mM AC, 4 mM GSH; (**C**)-C, 0.2 mM AC, 3 mM GSH; (**C**)-D, 0.3 mM AC, 2 mM GSH; (**C**)-E, 0.4 mM AC, 1 mM GSH; (**D**)-A, 0.1 mM AC, 1 mM Trolox; (**D**)-B, 0.15 mM AC, 0.8 mM Trolox; (**D**)-C, 0.2 mM AC, 0.6 mM Trolox; (**D**)-D, 0.3 mM AC, 0.4 mM Trolox; (**D**)-E, 0.4 mM AC; Trolox, 0.2 mM; (**E**)-A, 0.1 mM AC, 10 mM TEMPOL; (**E**)-B, 0.15 mM AC, 8 mM TEMPOL; (**E**)-C, 0.2 mM AC, 6 mM TEMPOL; (**E**)-D, 0.3 mM AC, 4 mM TEMPOL; (**E**)-E, 0.4 mM AC; 2 mM TEMPOL. Reaction time: 5, 20, and 40 min. * *p* < 0.05 with respect to additive interaction (IIC = 1) (one-tailed Student “*t*” test). Green asterisks indicate synergic interaction, red asterisks denote antagonistic interaction; the lack of an asterisk indicates additive interaction. AC, anthocyanins.

In the FRAP assay, the interactions of the extracts with most of the exogenous antioxidants studied oscillated around additivity (IIC = 1), with weak synergistic or antagonistic effects depending on the concentration ratio and reaction time. For ascorbic acid (Figure 3A), a weakly synergistic interaction was observed at the lowest extract/antioxidant ratio after 40 min reaction (IIC = 1.07 ± 0.02), but additive or weakly antagonistic in all other cases. For TEMPOL (Figure 3E), a weakly synergic interaction was found at the highest extract/antioxidant ratio for 5 min and 20 min reaction times (IIC of 1.09 ± 0.02 and 1.10 ± 0.02, respectively), with additivity of weak antagonism in all other cases. Additive or weakly antagonistic interactions were observed between the extract and gallic acid (Figure 3B) and between the extract and Trolox (Figure 3D). A clear-cut synergistic interaction between the extract and glutathione was observed across all conditions studied (Figure 3C).

The reaction rate ratios did not show any significant differences in the extract/antioxidant ratio for ascorbic acid and gallic acid. Still, they increased with the increasing extract/glutathione, extract/Trolox, and extract/TEMPOL ratios. For GSH and Trolox, the reaction rate ratios increased with increasing reaction time (Table S2). The correlation between reaction time and IIC in the FRAP assay was not significant for any antioxidant, as none of the correlation coefficients reached statistical significance (Table 4).

**Figure 4:**
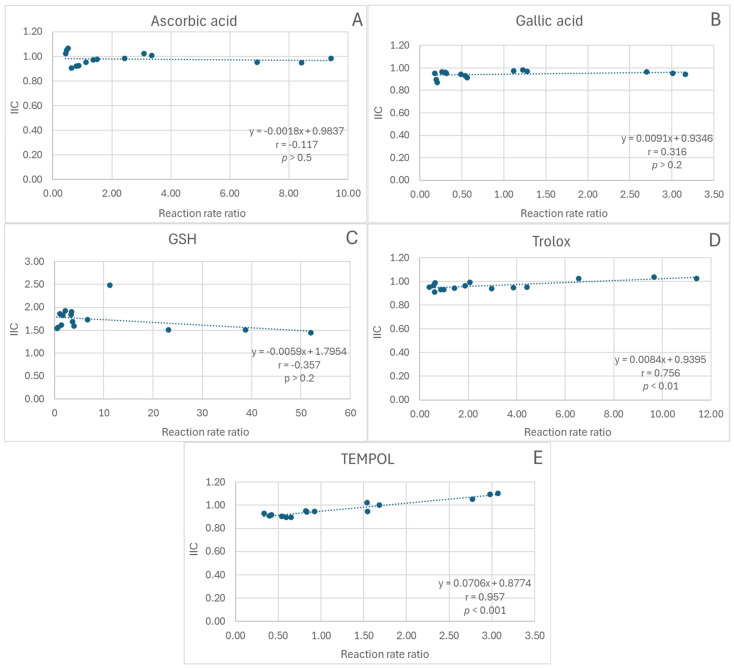
Dependence of the integrated interaction coefficient (IIC) on the reaction rate ratio in the FRAP assay. (**A**), red cabbage extract + ascorbic acid; (**B**), red cabbage extract + gallic acid; (**C**), red cabbage extract + glutathione; (**D**), red cabbage extract + Trolox; (**E**), red cabbage extract + TEMPO; r, Pearson correlation coefficient; *p*, statistical significance of the correlation coefficient.

In the FRAP assay, the dependence of IIC on the extract/antioxidant reaction rate ratio lacked statistical significance for ascorbic acid, gallic acid, and glutathione (Figure 4A–C) and was positive for Trolox and TEMPOL (statistical significance of correlation coefficients: *p* < 0.01 and *p* < 0.001, respectively; Figure 4D,E).

### 2.4. Sample Interaction Coefficient

Sample interaction coefficients (SICs) were calculated to examine the dependence of the IC on reagent concentration, as determined by the volume of introduced sample (1–6 µL). No systematic dependence of the SIC on the sample volume was found (Figures S1 and S2).

### 2.5. Summary of the Integrated Interaction Coefficient

Mean values calculated from IIC values averaged over all reaction times and extract/antioxidant ratio are summarized in Table 5. They confirm the significant antagonism between TEMPOL and the red cabbage extract in the ABTS^•^ decolorization assay and synergy between glutathione and the extract in the FRAP assay, and point to the overall weak antagonism in the interactions of ascorbic acid, gallic acid and glutathione with the extract in the ABTS^•^ decolorization assay, and of gallic acid and TEMPOL with the extract in the FRAP assay. In cases of weak antagonism, the antagonistic effect depended on the proportions of the reagents and the reaction time (Figure 2 and Figure 4).

Thus, it can be stated that, based on all assay conditions employed, in the ABTS decolorization assay, the interactions between the red cabbage extract and exogenous antioxidants were additive (Trolox), weakly antagonistic (0.90 ≤ IC < 1: ascorbic acid, gallic acid, and glutathione), or definitely antagonistic. In the FRAP assay, the interactions were additive (ascorbic acid and Trolox), weakly antagonistic (gallic acid and TEMPOL), and definitely synergic for glutathione.

## 3. Discussion

In this study, interactions between an aqueous red cabbage extract and exogenous antioxidants were examined in two popular antioxidant activity assays. The results for sample interaction coefficient (SIC) showed variability dependent on the reaction time but no systematic dependence on the concentration of reagents in the concentration range studied (final concentrations in the reaction mixture: extract, 0.2–4.7 µM in anthocyanins, 1.9–46.6 µM ascorbic acid, 0.5–11.7 µM gallic acid, 1.0–23.3 µM glutathione, 1.8–41.9 µM Trolox, 49.8–1165.1 µM TEMPOL in the ABTS^•^ decolorization assay and extract, 0.5–11.7 µM in anthocyanins, 1.0–29.1 µM ascorbic acid, gallic acid and Trolox, 5.0–145.6 µM glutathione, and 10.0–291.3 µM TEMPOL in the FRAP assay). However, in cases when most results indicated additive interactions, statistically significant synergy or antagonism was found in some cases (without any systematic dependence). Deviations from additivity (SIC ≠ 1) classified as statistically significant by the Student “*t*” test were especially frequent for the lowest volumes of the reagents added (1 µL), where the pipetting error was maximal. It highlights the danger of erroneous conclusions when drawing conclusions on the type of interaction based on SIC. Therefore, we prefer to use the integrated interaction coefficient (IIC), which, by interpolating the concentration dependence of the reaction with an indicator, essentially eliminates errors in estimating individual SICs. The IIC values were also dependent on reaction time and the reagent concentration ratio, but were generally closer to additivity in most cases. No general relationship was evident from studies of the dependence of the IIC on the extract/antioxidant reaction rate ratio.

No clear picture emerges from the literature on interactions between antioxidants in antioxidant activity assays, particularly regarding their dependence on antioxidant concentration and the ratio of components in the mixtures. Ilyasov et al. studied the interaction of flavonoids (taxifolin, quercetin, rutin, and morin), finding weak antagonistic effects in the ABTS^•^ decolorization assay when using the same method of analysis as that employed in the present study, and mostly weak synergistic effects when analyzing the data using Webb’s simulation. The amount of ABTS^•^ scavenged by one molecule of all antioxidants studied (n-value) increased during reaction time from 1 to 10, 20, and 30 min [10], in agreement with our findings for various antioxidants [28]. Synergic interactions between chlorogenic acid and gallic acid and between caffeic and gallic acid were found in the ABTS^•^ decolorization assay [29].

No regularity was observed in the interactions between phenolic acids in binary systems in the FRAP method when the concentrations of reagents were increased from 3.2 to 16.1 and 32.3 µM in the reaction medium. Still, the interaction coefficient tended to deviate less from additivity as the antioxidant concentrations increased [7].

In the catechin + quercetin and catechin + quercetin-3-β-glucoside systems, synergy was observed; its magnitude has an increasing tendency with decreasing proportion of catechin, when the proportion of the components increased from 1:3 to 3:1. However, no such regularity was observed in other binary flavonoid systems [14]. Another study reported a fairly regular increase in the synergy of interaction of *Gingko biloba* procyanidins with Gingko flavone, organic acids, and ginkgolide, along with an increasing proportion of Gingko flavone in the mixture in the ABTS^•^ decolorization and DPPH^•^ decolorization assays. Still, no such regularity was observed in the interaction of organic acids with ginkgolide [30].

The interactions among kaempferol, quercetin, and myricetin were mainly antagonistic in the FRAP and ABTS^•^ assays decolorization assays; the interactions were also concentration-dependent [31]. Interaction of curcumin with (−)-epicatechin or with the epicatechin fraction from green tea showed synergy in the ABTS^•^ decolorization assay, the most substantial effect being found for the equimolar mixture of pure compounds [15]. Interactions between various phenolics (catechin, chlorogenic acid, cyanidin, cyanidin 3-glucoside, cyanidin 3-rutinoside, epicatechin, peonidin, peonidin 3-glucoside, quercetin, quercetin 3-glucoside, quercetin 3-galactoside and quercetin 3-rutinoside) in the ABTS^•^ decolorization assay were mostly additive, although in some cases, the effects were weakly synergic or antagonistic; the deviations from the additivity did not exceed several percent [32].

The synergic interaction between the extract and glutathione was observed in this study in the FRAP assay but not in the ABTS^•^ decolorization assay (Table 5). Similar effects, i.e., different types of interactions across various assays, have been reported for other systems. e.g., the interaction between quercetin and caffeic acid was additive in the FRAP and DPPH^•^ decolorization assays but antagonistic in the ABTS^•^ decolorization assay. In contrast, the interaction between resveratrol and caffeic acid was synergic in the FRAP assay and antagonistic in the DPPH^•^ decolorization and ABTS^•^ decolorization assays, evidently due to differences in the reaction mechanisms of the antioxidant assays [33]. The interaction between the *Gingko biloba* gingkolide and organic acids was synergic in the DPPH^•^ decolorization assay for all proportions of the reagents studied (from 1:9 to 9:1) but mostly antagonistic in the ABTS^•^ decolorization assay [30].

In a study of interactions between trehalose and ascorbic acid, catechin, gallic acid and quercetin, an antagonistic interaction was revealed, with the interaction coefficient decreasing with decreasing concentrations of the antioxidants [34]. However, interpretation of these results is not straightforward, since trehalose did not reduce DPPH^•^ at all, so the effect cannot be referred to as an interaction between antioxidants but rather as due to other factors.

Interactions between complex food products were also studied. For example, interaction of spirulina with apple juice was found to be additive in the FRAP assay and antagonistic in the ABTS^•^ decolorization assay; interaction of spirulina and Japanese quince syrup was synergic in the FRAP assay and additive in the ABTS^•^ decolorization assay; interaction between spirulina and cranberry syrup was synergic in the FRAP assay and antagonistic in the ABTS^•^ decolorization assay [35].

Interactions between essential oils of *Laurus nobilis*, *Lavandula stoechas* and *Mentha pulegium* in the ABTS^•^ decolorization assay were mainly synergic but additive or antagonistic in some cases, depending on the proportions of the oils and method of assay [36]. ABTS^•^ decolorization assay demonstrated synergic interaction between green tea, honey and *Citrus limonum* extract [37]. Analysis of interactions between various food products using the QUENCHER procedure [38] and ABTS^•^ decolorization assay demonstrated antagonistic, additive and synergic interactions between multiple products and often changes in the type of interaction after simulated gastric, intestinal, and colonic digestion [39].

**Figure 5:**
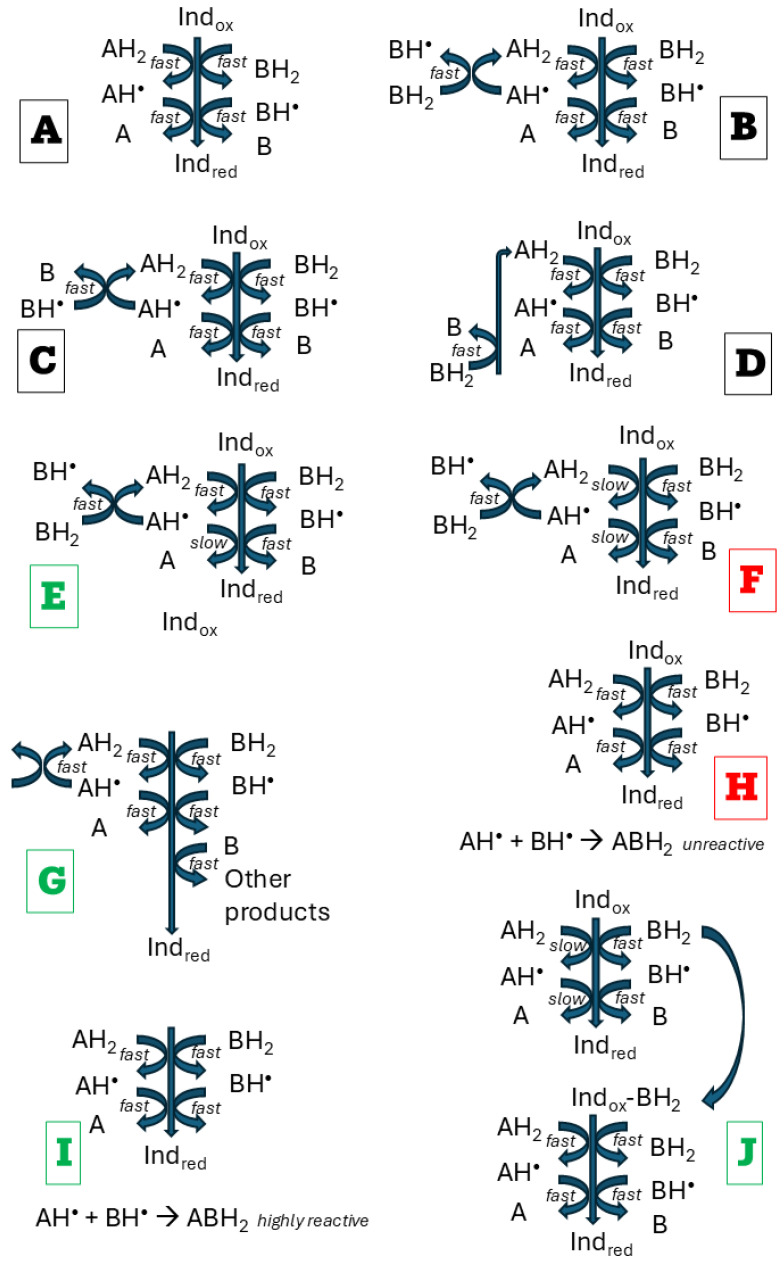
The main possible effects of interactions between antioxidants undergoing two-step oxidation: maintenance of additivity (black), synergy (green), and antagonism (red). Ind_ox_, oxidized indicator; Ind_red_, reduced indicator. (**A**), no interaction between antioxidants AH_2_ and BH_2_; (**B**,**C**), regeneration antioxidant AH_2_ by reduction of one-electron oxidation products of AH_2;_ (**D**), regeneration of AH_2_ by reduction of two-electron oxidation product of AH_2_ by BH_2_; (**E**), regeneration of antioxidant AH_2_ from its weakly reactive one-electron oxidation product of leads to synergy; (**F**), consumption of an antioxidant reacting with the indicator for the regeneration of another antioxidant weakly reactive with the indicator leads to antagonism; (**G**), faster consumption of BH_2_ due to regeneration of AH_2_ enables a greater participation of still reactive B in the reduction of the indicator, leading to synergy; (**H**,**I**), reaction between one-electron oxidation products of both antioxidants leads to antagonism if the reaction product is not reactive (**H**) or synergy if the product is hyperreactive (**I**,**J**), complexing the indicator by one antioxidant facilitates the reduction of the indicator by another antioxidant.

It should be kept in mind that even an apparently simple chemical reaction usually involves many intermediate steps, one of which is a bottleneck that governs the reaction rate. Intermediates at this critical step may have a decisive effect on the reaction outcome, and without detailed knowledge of the reaction mechanism, its course is challenging to explain [40]. Nevertheless, partial explanations are possible (Figure 5). If two antioxidants do not interact, additivity is observed (Figure 5A). In homogenous systems, the most plausible explanation for synergy is the regeneration of one antioxidant by another [13,41,42,43]. However, not always regeneration of one antioxidant by another may result in synergy. Suppose both antioxidants A and B, and their one-electron oxidation products, react rapidly (within the time of the assay) with the indicator. In that case, regeneration of one antioxidant by another does not change the number of reactive reducing species in the system, and additivity is maintained (Figure 5B–D). When the product of one-electron oxidation of an antioxidant shows low reactivity, and the reduction of the indicator is mainly dependent on the first one-electron reaction of this oxidant, regeneration of this antioxidant from its one-electron oxidation product by another antioxidant will result in synergy (Figure 5E). Conversely, consumption of a fraction of a reactive antioxidant for regeneration of a weakly reactive antioxidant will result in antagonism (Figure 5F). Enhanced utilization of one antioxidant due to regeneration of another one may lead to synergy when the two-electron oxidation product of this antioxidant can further reduce the indicator (Figure 5G). Reaction between one-electron oxidation products of both antioxidants can result in antagonism when the reaction product does not react with the indicator (Figure 5H) or synergy when the product is more reactive (reacts with a greater number of molecules of the indicator) than the individual one-electron oxidation products (Figure 5I). One antioxidant can form a complex with the indicator, facilitating its reduction by another antioxidant (Figure 5J).

In any case, elucidation of the mechanisms of interaction would be possible based on analyses of the reaction mixture composition and the intermediates and final products formed.

In the present study, the only clear-cut deviations from additivity of interaction were observed in the systems of extract-TEMPOL, showing an antagonistic effect in the ABTS^•^ decolorization assay, and extract-glutathione, exhibiting a synergic effect in the FRAP assay. The mechanisms of both assays differ: FRAP is a SET assay, and ABTS^•^ decolorization is a SET-HAT assay [44,45,46]. Variant oxidants used in both assays (ABTS^•^ and Fe^3+^, respectively).

These deviations from additivity appear to result from distinct mechanisms. Among the endogenous antioxidants of red cabbage extract, polyphenols, including anthocyanins, are predominant (Table 1 and Table 2). In the ABTS^•^ decolorization assay, they reduce ABTS^•^, producing semiquinone radicals. A semiquinone radical can also reduce ABTS^•^, forming a quinone. One-electron oxidation of TEMPOL by ABTS^•^ leads to the formation of an oxoammonium cation, which is a strong oxidant (E_o_′ of 0.81 V) [47]. Redox potentials of semiquinones cover a broad range of values, depending on substituents, but they are lower than 0.8 V [48]. In the presence of TEMPO oxoammonium cation, a fraction of hydroquinones and semiquinone radicals can be oxidized to semiquinone radicals and quinones, respectively. They cannot reduce ABTS^•^, decreasing the yield of ABTS^•^ reduction. In this reaction, TEMPO is regenerated, but it reacts at a lower rate with ABTS^•^ than semiquinone radicals, and the rate of ABTS^•^ reduction is lowered, which accounts for the antagonistic effect:

ABTS^•^ + H_2_Q → ABTS + HQ^•^ + H^+^ (fast)

ABTS^•^ + HQ^•^ → ABTS + Q + H^+^ (fast)

ABTS^•^ + TEMPOL^•^ → ABTS + TEMPOL^+^ (slow)

TEMPOL^+^ + H_2_Q → TEMPOL^•^ + HQ^•^

TEMPOL^+^ + HQ^•^ → TEMPOL^•^ + Q,

where H_2_Q is a hydroquinone, Q is quinone, HQ^•^ is the semiquinone radical, TEMPOL^•^ underscores the free radical nature of TEMPOL, and TEMPOL^+^ is the oxoammonium cation of TEMPOL. This situation corresponds to the case presented in Figure 5F. In fact, the reaction is more complicated, including the formation of transient covalent adducts of radical oxidation products of polyphenols with ABTS^•^ [49].

Glutathione is weakly reactive in ferric ion reduction. The synergistic interaction of glutathione with the extract in the FRAP assay can be suggested to be due to the facilitation of this reaction by some components of the extract (e.g., chelation of iron, which may decrease its redox potential) [50] (Figure 5J) or formation of glutathione-polyphenol conjugates that may be more active in Fe^3+^ reduction than glutathione itself [51,52] (Figure 5I). Such a synergistic effect is specific for Fe^3+^ so it could not be observed in the ABTS^•^ decolorization assay.

In vitro assays provide limited information about the reactivity and interactions of antioxidants. The results of such assays cover phenomena occurring within a limited time frame and environment and do not account for slow oxidation reactions or side reactions with other components in more complex systems. Thus, the validity of extrapolating conclusions from in vitro assays to real systems must be approached cautiously. Interactions with the matrix, as well as conditions of gastrointestinal digestion, may affect interactions between food antioxidants and warrant further study.

However, in some cases, such data allow for meaningful predictions. As far as the present results are concerned, the synergism between glutathione and the red cabbage extract may indicate that glutathione facilitates Fe^3+^ reduction. Although glutathione reactivity for these ions is low, it can be considerably enhanced by other food components.

## 4. Materials and Methods

### 4.1. Reagents, Materials and Equipment

2,2′-Azino-bis (3-ethylbenzothiazoline-6-sulfonic acid) (ABTS; CAS no. 504-14-6; cat. no. 10102946001; purity ≥ 99%) was provided by Roche (Warsaw, Poland). Iron(III) chloride (FeCl_3_; CAS no. 7705-08-0; cat. no. 451649; purity ≥ 99.99%) was obtained from MedChemExpress (Monmouth Junction, NJ, USA). The Folin–Ciocalteu phenol reagent (cat. no. F9252), 2,4,6-Tri(2-pyridyl)-s-triazine (TPTZ; CAS no. 3682-35-7; cat. no. T1253), gallic acid monohydrate (CAS no. 5995-86-8; cat. no. 398225), sodium ascorbate (CAS no. 134-03-2, cat. no. 11140), Trolox (CAS no. 53188-07-1, cat. no. 648471), sodium carbonate (CAS no. 497-19-8, cat. no. 106392), potassium persulfate (CAS no. 7727-21-1, cat. no. 216224), ascorbic acid (CAS no. 50-81-7, cat.no. A7506), glutathione, (CAS no. 70-18-8; cat. no. Y0000517), TEMPOL (CAS no. 2226-96-2, cat. no. 176141), quercetin (CAS no. 117-39-5, cat. no. Q4951-10G), sinigrin hydrate (CAS no. 3952-98-5, cat. no. 85440), dimethyl sulfoxide (DMSO) (CAS no. 67-68-5, cat. no. D2438), hydrochloric acid 37% (CAS no. 7647-01-0, cat. no. 320331), aluminum chloride (CAS no. 7446-70-0, cat. no. 206911, sodium tetrachloropalladate (CAS no. 13820-53-6, cat. no. 379808) and potassium persulfate (CAS no. 7727-21-1, cat. no. 216224), were provided by Merck (Poznan, Poland). Sodium dihydrogen phosphate (CAS no. 10049-21-5; cat. no. PM306.500, purity 98–103%), and sodium hydrogen phosphate (CAS no. 7782-85-6; cat. no. SPD579.1, purity 98–102%) produced by BioShop Canada Inc. (Burlington, ON, Canada) were purchased from Lab Empire (Rzeszow, Poland). Glacial acetic acid CAS no. 64-19-7; cat. no. JT9522-2) and sodium acetate anhydrous (CAS no. 127-09-3; cat. no. BN60/6191; purity ≥ 99%) were obtained from Avantor Performance Materials (Gliwice, Poland). Phenol (CAS no. 108-95-2, cat. no. 05168) and glucose anhydrous (CAS no. 50-99-7, cat. no 459560117) were provided by P.O.Ch. (Gliwice, Poland). Methanol (CAS no. 67-56-1, cat. no. 34860) was bought from Honeywell (Warsaw, Poland). Sodium hydroxide (CAS no. 1310-73-2) was from Warchem (Zakręt, Poland). Hydrogen peroxide (30%; CAS no. 7722-84-1, cat. no. 118851934) and sulfuric acid 98% (CAS no. 7664-93-9, cat. no. 115750013) was provided by Chempur (Piekary Śląskie, Poland).

Red cabbage (*Brassica oleracea* var. *capitata* f. *alba* and f. *rubra*), grown in the continental agroclimatic zone, was purchased in a local supermarket in Rzeszów.

Distilled water was purified using a Milli-Q system (Millipore, Bedford, MA, USA). Transparent flat-bottom 96-well plates (cat. no. 655101) (Greiner, Kremsmünster, Austria) were used for the assays. Absorptiometric measurements were performed in a Spark (Ref. 30086376) multimode microplate reader (Tecan Group Ltd., Männedorf, Switzerland).

### 4.2. Preparation and Description of Red Cabbage Extract

External leaves of the cabbage head were removed, the cabbage was washed and chopped into about 1 cm × 1 cm × 1 cm fragments, ground in a mortar with 100 mM acetate buffer, pH 5.0 (1 g leaves/9 mL of water), shaken for 30 min, and centrifuged (3000× *g*, 20 min). The supernatant was aliquoted and stored frozen at −80 °C until analysis (for no more than 1 month).

### 4.3. Estimation of Polyphenol Concentration

The phenolic concentration in the extract was estimated using the Folin–Ciocalteu reagent [53]. Briefly, 25 µL of the diluted extract was added to 125 µL of 1 M Folin–Ciocalteu reagent in wells of a 96-well plate. After 4 min, 100 µL of saturated disodium carbonate solution (about 75 g/L) was added. The absorbance of the mixture was measured at 750 nm after incubation for one hour at ambient temperature. The absorbance reading was compared with the standard curve prepared with gallic acid and expressed as gallic acid equivalents/L of the extract.

### 4.4. Estimation of Flavonoid Concentration

The protocol described by Sultana et al. [54] was followed in a slight modification. Diluted extract (100 µL) was added with the same volume of 10% aluminum chloride. After 30 min of incubation, the absorbance was read against a reagent blank. The standard curve was prepared with quercetin, so the results are expressed in quercetin equivalents.

### 4.5. Estimation of Anthocyanin Concentration

Estimation of anthocyanin content was performed according to a slightly modified procedure of Lee et al. [55]. Briefly, 125 µL aliquots of the extract were added with 875 µL of 0.1 M acetate buffer, pH 4.5 or 1.5 M HCl, and the absorbance of both samples was measured at the absorption maximum (at about 520 nm) and 700 nm.

The anthocyanin concentration c was calculated as:

c [µmol/L] = A × dilution × 10^3^)/(ε × l), where

A = (A − A_700nm_)_pH1_ − (A − A_700nm_)_pH4.5_,

where MW, molecular weight; ε, molar absorption coefficient for kuromarin (ε = 26,900 M^−1^ cm^−1^); l, length of the optical path.

### 4.6. Estimation of Total Carbohydrate Concentration

Total carbohydrate concentration was estimated using the phenol-sulfuric acid method [56] in a slight modification. In brief, 400 µL of a diluted extract was added with 10 µL of 80% phenol and 1 mL of concentrated sulfuric acid. After 15 min, the absorbance of the solution was read at 490 nm against a reagent blank containing no extract, subtracting the absorbance of a blank containing the colored extract and no phenol. A standard curve was prepared with glucose, and the results are expressed in µmol glucose equivalents/L.

### 4.7. Estimation of Glucosinolate Concentration

Total glucosinolate concentration was estimated using sodium tetrachloropalladate according to the protocol described by Mawlong et al. [57], with sinigrin as the standard. Briefly, 50 µL of the extract was added to 1500 µL of 2 mM sodium tetrachloropalladate. The samples were incubated for 60 min, and the absorbance was measured at 425 nm against a reagent blank. Sinigrin was used to obtain the standard curve.

### 4.8. Analysis of Polyphenolic Compounds Using UPLC-PDA-ESI-MS/MS

Polyphenolic compounds were analyzed using a UPLC-PDA-ESI-MS/MS Waters ACQUITY system (Waters, Milford, MA, USA) consisting of a binary pump manager, sample manager, column manager, photodiode array (PDA) detector, and tandem quadrupole mass spectrometer (TQD) with electrospray ionization (ESI), following the method described previously [58]. The separation was performed on a BEH C18 column (100 mm × 2.1 mm i.d., 1.7 m, Waters) maintained at 50 °C. For the anthocyanins investigation, the following solvent system was applied: mobile phase A (2% formic acid in water, *v*/*v*) and mobile phase B (2% formic acid in 40% acetonitrile in water, *v*/*v*). For other polyphenolic compounds, a lower concentration of formic acid was used (0.1%, *v*/*v*). The gradient program was set as follows: 0 min, 5% B; from 0 to 8 min, linear to 100% B; and from 8 to 9.5 min, for washing and returning to initial conditions. The flow rate was 0.35 mL/min. The following parameters were used for TQD: capillary voltage 3.5 kV, cone voltage, 30 V in positive and negative mode; the source was kept at 120 °C, and the desolvation temperature was 350 °C, con gas flow 100 L/h, and desolvation gas flow 800 L/h. Argon was used as the collision gas at a flow rate of 0.3 mL/min. Polyphenolic detection and identification were based on specific PDA spectra, mass-to-charge ratios, and fragment ions obtained after collision-induced dissociation (CID). Quantification was achieved by the injection of solutions of known concentrations that ranged from 0.05 to 5 µg/mL (R^2^ of 0.9998) of phenolic compounds as standards. All of the determinations were performed in duplicate and expressed as µg/mL. Waters MassLynx software v.4.1 (Waters, Milford, MA, USA) was used for data acquisition and processing.

### 4.9. ABTS^•^ Decolorization Assay

A modification [28] of the ABTS^•^ decolorization assay [59] was employed. Briefly, various volumes (1–6 µL) of solutions of red cabbage extract and/or an antioxidant were introduced to wells of a 96-well microplate, each pre-filled with 200 µL of ABTS^•^ solution of absorbance 1.0 (at 734 nm) in a well of a 96-well microplate. The stock ABTS^•^ solution was prepared by overnight oxidation of 7 mM ABTS with 2.45 mM potassium persulfate (final concentrations). This stock solution was diluted in phosphate-buffered saline (PBS; 145 mM NaCl in 10 mM sodium phosphate, pH 7.4).

The concentrations of the antioxidants in solutions introduced to the wells were: extract 0.04 mM in anthocyanins, 1.6 mM ascorbic acid; extract, 0.08 mM in anthocyanins, 1.2 mM ascorbic acid; extract, 0.12 mM in anthocyanins, 0.8 mM ascorbic acid; extract, 0.12 mM in anthocyanins, 0.4 mM ascorbic acid; extract 0.04 mM in anthocyanins, 0.4 mM gallic acid; extract, 0.08 mM in anthocyanins, 0.3 mM gallic acid; extract, 0.12 mM in anthocyanins, 0.2 mM gallic acid; extract, 0.16 mM in anthocyanins, 0.1 mM gallic acid; extract 0.04 mM in anthocyanins, 0.8 mM GSH; extract, 0.08 mM in anthocyanins, 0.6 mM GSH; extract, 0.12 mM in anthocyanins, 0.4 mM GSH; extract, 0.16 mM in anthocyanins, 0.2 mM GSH; extract 0.04 mM in anthocyanins, 1.44 mM Trolox; extract, 0.08 mM in anthocyanins, 1,08 mM Trolox; extract, 0.12 mM in anthocyanins, 0.72 mM Trolox; extract, 0.16 mM in anthocyanins, 0.36 mM Trolox; extract 0.04 mM in anthocyanins, 40 mM TEMPOL; extract, 0.08 mM in anthocyanins, 30 mM TEMPOL; extract, 0.12 mM in anthocyanins, 20 mM TEMPOL; extract, 0.16 mM in anthocyanins, 10 mM TEMPOL. The samples were incubated on a shaker. The drop in absorbance after 1-, 30-, and 60-min incubations at ambient temperature (21 ± 1 °C), corrected for the absorbance decrease in a blank sample containing ABTS^•^ solution without any additive, was used as a measure of antioxidant activity.

**Figure 6:**
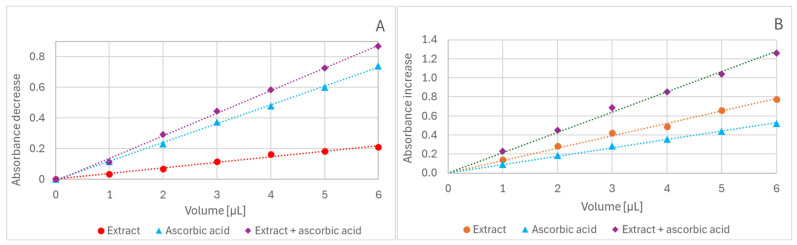
Dependence of ABTS^•^ decolorization (**A**) and absorbance increase in the FRAP assay (**B**) on the volume of added ascorbate solution (1.6 mM), extract (0.2 mM in anthocyanins), and ascorbate solution + extract. Reaction time: 60 min.

The amounts of red cabbage extract and antioxidants were chosen to provide appropriate levels of reduction and stay in the range of linear dependence of the extent of reduction on the volume of antioxidants, so that the extent of ABTS^•^ decolorization did not exceed 90% (Figure 6A).

### 4.10. Ferric Reducing Antioxidant Power (FRAP) Assay

The method proposed by Benzie and Strain [60] was slightly modified. Briefly, in-creasing volumes (1–6 µL) of red cabbage extract, an antioxidant, or red cabbage extract and an antioxidant were added to wells of a 96-well microplate pre-filled with 200 μL of the working solution composed of 0.3 M acetate buffer, pH 3.6 (10 volumes), 10 mM TPTZ in 40 mM HCl (1 volume) and 20 mM FeCl_3_ (1 volume), prepared immediately before use. The concentrations of the antioxidants in solutions introduced to the wells were: extract 0.1 mM in anthocyanins, 1 mM ascorbic acid; extract, 0.15 mM in anthocyanins, 0.8 mM ascorbic acid; extract, 0.2 mM in anthocyanins, 0.6 mM ascorbic acid; extract, 0.3 mM in anthocyanins, 0.4 mM ascorbic acid; extract, 0.4 mM in anthocyanins; extract, 0.1 mM in anthocyanins, 1 mM gallic acid; extract, 0.15 mM in anthocyanins, 0.8 mM gallic acid; extract, 0.2 mM in anthocyanins, 0.6 mM gallic acid; extract, 0.3 mM in anthocyanins, 0.4 mM gallic acid; extract, 0.4 mM in anthocyanins, 0.2 mM gallic acid; extract 0.1 mM in anthocyanins, 5 mM GSH; extract, 0.15 mM in anthocyanins, 4 mM GSH; extract, 0.2 mM in anthocyanins, 3 mM GSH; extract, 0.3 mM in anthocyanins, 2 mM GSH; extract, 0.4 mM in anthocyanins, 1 mM GSH; extract 0.1 mM in anthocyanins, 1 mM Trolox; extract, 0.15 mM in anthocyanins, 0.8 mM Trolox; extract, 0.2 mM in anthocyanins, 0.6 mM Trolox; extract, 0.3 mM in anthocyanins, 0.4 mM Trolox; extract, 0.4 mM in anthocyanins, 0.2 mM Trolox; extract 0.1 mM in anthocyanins, 10 mM TEMPOL; extract, 0.15 mM in anthocyanins, 8 mM TEMPOL; extract, 0.2 mM in anthocyanins, 6 mM TEMPOL; extract, 0.3 mM in anthocyanins, 4 mM TEMPOL; extract, 0.4 mM in anthocyanins, 2 mM TEMPOL. After incubation for 5, 20, and 60 min on a shaker at ambient temperature, the absorbance of the Fe^2+^-TPTZ complex was measured at 593 nm.

A linear dependence of the absorbance increase on the concentration of red cabbage extract and antioxidants was observed in the FRAP assay (Figure 6B). The antioxidant concentrations were chosen to provide activity comparable to that of the extract.

### 4.11. Interactions Between Red Cabbage Extract and Antioxidants in Antioxidant Activity Assays

From the slope of the dependence of absorbance decrease (ABTS^•^ decolorization assay) or increase (FRAP) on the amount of extract, an antioxidant, or both, integrated antioxidant activity was calculated as shown in [61]. The integrated interaction coefficient IIC was determined as

IIC = (integrated antioxidant activity of the extract + an antioxidant)/[(integrated antioxidant activity of the extract) + (integrated antioxidant activity of the antioxidant)]

The sample interaction coefficient was also calculated for individual sets of samples:

SIC = (antioxidant activity of x µL of the extract + x µL of an antioxidant)/[(antioxidant activity of x µL of the extract) + (antioxidant activity of x µL of the antioxidant)]

**Figure 7:**
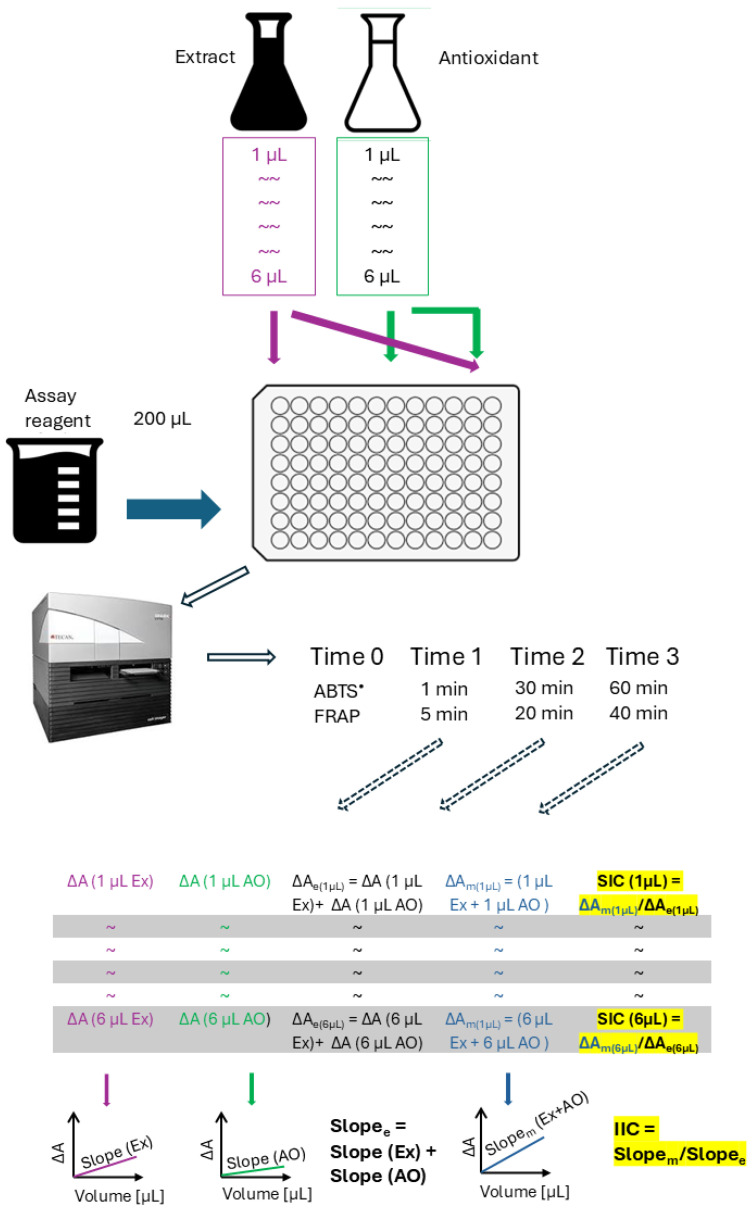
Scheme of the experiment and calculations. Index _e_ denotes expected, and index _m_ measured values; ΔA, absorbance change; SIC, sample interaction coefficient; IIS, integrated interaction coefficient.

The scheme of the experiment and the principle of calculation of the sample interaction coefficient (SIC) and integrated interaction coefficient (IIC) are shown in Figure 7.

IIC or SIC of 1 indicates an additive interaction, IIC or SIC < 1 an antagonistic interaction, and IIC or SIC > 1 a synergistic interaction. We classified interaction coefficient values between 0.90 and 1.0 as indicative of weak antagonism, and those between 1.0 and 1.1 as weakly synergic, since, despite the formal statistical significance of the difference from 1, the practical importance of such antagonism or synergism, respectively, seems doubtful.

### 4.12. Ratio of Reaction Rates

The ratio of reaction rates of the extract and an antioxidant used was calculated as the ratio of absorbance decrease (in the ABTS decolorization assay) or absorbance increase (in the FRAP assay), corrected for absorbance changes in respective blank samples, for the extract and an antioxidant, respectively.

### 4.13. Statistics

All experiments were conducted on three batches of the extract. The results are presented as arithmetic means ± SD. Statistical significance of differences in SIC and IIC from the value of 1 (indicating additive interaction) was estimated using the one-tailed Student “*t*” test. The Pearson correlation coefficient was used to estimate the strength of the association between variables. Values of *p* < 0.05 were considered statistically significant. The calculations were performed in Excel (Microsoft 365; Redmond, WA, USA).

## 5. Conclusions

Results of the present study confirm that the type of interaction between antioxidants may differ across methods of antioxidant activity assay. Moreover, they demonstrate that the formal type of interaction may depend on the time of reaction and the ratio of antioxidants within a single assay, and may be influenced by stochastic factors related to pipetting accuracy. Therefore, the conclusion on the type of interaction should be based on a series of assays covering a range of conditions to demonstrate unequivocally a deviation from additivity.

## Figures and Tables

**Table 1:** The chemical components of the red cabbage extract.

Components	Mean ± SD
Polyphenols [µmol gallic acid equivalents/L]	1315 ± 40
Flavonoids [µmol quercetin equivalents/L]	588 ± 49
Anthocyanins [µmol kuromarin equivalents/L]	408 ± 24
Total carbohydrates [µmol glucose equivalents/L]	41.7 ± 0.1
Glucosinolates [µmol sinigrin equivalents/L]	3.76 ± 0.14

**Table 2:** Profile and content of individual phenolic compounds identified by UPLC-PDA-MS/MS in red cabbage extract.

Compound	Rt [min]	λ_max_ [nm]	[M-H]^+/−^ m/z		Content [µg/mL]
			MS	MS/MS	
*Anthocyanins*					
Cyanidin 3-*O*-sophoroside-5-*O*-glucoside	2.01	279,512	773^+^	611,449,287	73.93 ± 0.04
Cyanidin 3-*O*-sophoroside	2.14	276,514	611^+^	287	2.90 ± 0.07
Cyanidin 3-*O*-glucoside	2.42	276,515	449^+^	287	2.04 ± 0.02
Cyanidin 3-*O*-(sinapoyl)-sophoroside-5-*O*-glucoside isomer I	2.74	281,527	979^+^	817,449,287	37.51 ± 0.42
Cyanidin 3-*O*-(caffeoyl)(*p*-coumaroyl)-sophoroside-5-*O*-glucoside	3.08	279,522	1081^+^	919,449,287	7.27 ± 0.00
Cyanidin 3-*O*-(feruloyl)-trigluoside-5-*O*-glucoside	3.22	281,522	1111^+^	949,787,449,287	4.46 ± 0.13
Cyanidin 3-*O*-(caffeoyl)(sinapoyl)-sophoroside-5-*O*-glucoside	3.27	281,524	1141^+^	979,449,287	5.45 ± 0.02
Cyanidin 3-*O*-(caffeoyl)(feruloyl)-sophoroside-5-*O*-glucoside	3.69	279,534	1111^+^	949,449,287	6.68 ± 0.02
Cyanidin 3-*O*-(sinapoyl)-sophoroside-5-*O*-glucoside isomer II	3.76	281,536	979^+^	817,449,287	5.13 ± 0.06
Cyanidin 3-*O*-(caffeoyl)-sophoroside-5-O-glucoside	3.82	279,536	935^+^	773,449,287	4.09 ± 0.10
Cyanidin 3-*O*-(*p*-coumaroyl)-sophoroside-5-O-glucoside	4.14	279,524	919^+^	757,449,287	7.09 ± 0.50
Cyanidin 3-*O*-(sinapoyl)-sophoroside-5-*O*-glucoside isomer III	4.26	279,524	979^+^	817,449,287	20.78 ± 0.19
Cyanidin 3-*O*-(*p*-coumaroyl)(sinapoyl)-sophoroside-5-*O*-glucoside	4.65	279,534	1125^+^	963,449,287	9.25 ± 0.31
Cyanidin 3-*O*-(feruloyl)(sinapoyl)-sophoroside-5-*O*-glucoside	4.75	279,534	1155^+^	993,449,287	6.04 ± 0.07
Cyanidin 3-*O*-(sinapoyl)(sinapoyl)-sophoroside-5-O-glucoside	4.84	279,536	1185^+^	1,023,449,287	42.82 ± 0.85
*Other phenolics*					
*di*-Sinapoyl-*di*-glucoside	2.36	333	789^−^	627,609,447,223	6.57 ± 0.15
*di*-Sinapoyl-coumaroyl-*tri*-glucoside isomer I	3.18	307	1097^−^	935,609,447,223	0.90 ± 0.01
*di*-Sinapoyl-feruloyl-*tri*-glucoside	3.34	331	1127^−^	965,609,447,137	14.74 ± 0.16
*tri*-Sinapoyl-*tri*-glucoside	3.45	305	1157^−^	995,609,447,223	1.19 ± 0.00
*di*-Sinapoyl-coumaroyl-*tri*-glucoside isomer II	3.53	315	1097^−^	935,609,447,223	2.21 ± 0.06
*tri*-Sinapoyl-*di*-glucoside isomer I	3.63	322	995^−^	815,447,223	1.50 ± 0.00
1-*O*-Sinapoyl-glucoside	3.79	324	385^−^	223	1.79 ± 0.01
*di*-Sinapoyl-coumaroyl-*di*-glucoside	4.24	315	935^−^	773,447,223	1.55 ± 0.02
3,4-*O*-*di*-Caffeoylquinic acid	4.41	324	515^−^	353,191	2.65 ± 0.07
*di*-Sinapoyl-feruloyl-*di*-glucoside	4.50	324	965^−^	785,447,223	2.91 ± 0.20
*tri*-Sinapoyl-*di*-glucoside isomer II	4.60	326	995^−^	815,447,223	4.76 ± 0.04
*tri*-Sinapoyl-*di*-glucoside isomer III	4.93	327	995^−^	815,447,223	5.50 ± 0.18
*tri*-Sinapoyl-*tri*-glucoside	5.47	321	1121^−^	897,223	4.73 ± 0.06
Sinapoyl-*di*-coumaroyl-*tetra*-glucoside	5.69	331	1183^−^	993,223	31.06 ± 0.62
*tri*-Sinapoyl-*di*-glucoside	7.42	326	959^−^	735,223	6.05 ± 0.25
TOTAL					323.5 ± 2.27

**Table 3:** Pearson correlation coefficients for the dependence of IIC on the reaction time in the ABTS^•^ decolorization assay (n = 12).

Antioxidant	r	Statistical Significance
Ascorbic acid	0.005	*p* > 0.5
Gallic acid	−0.684	***p* < 0.02**
GSH	−0.572	*p* > 0.05
Trolox	−0.702	***p* < 0.02**
TEMPOL	−0.839	***p* < 0.001**

**Table 4:** Pearson correlation coefficients for the dependence of IIC on the reaction time in the FRAP assay (n = 15).

Antioxidant	r	Statistical Significance
Ascorbic acid	0.246	*p* > 0.2
Gallic acid	−0.416	*p* > 0.1
Glutathione	−0.176	*p* > 0.5
Trolox	0.262	*p* > 0.2
TEMPOL	−0.185	*p* > 0.5

**Table 5:** Average values of IIC for all reaction times and extract/antioxidant ratios measured (mean ± SD); n = 12 in the ABTS^•^ decolorization assay and n = 15 for the FRAP assay.

Antioxidant	ABTS^•^ Decolorization Assay	FRAP Assay
Ascorbic acid	0.94 ± 0.03 *	0.98 ± 0.05
Gallic acid	0.90 ± 0.07 *	0.94 ± 0.03 *
GSH	0.91 ± 0.07 *	1.73 ± 0.26 *
Trolox	0.98 ± 0.07	0.97 ± 0.07
TEMPOL	0.66 ± 0.22 *	0.97 ± 0.04 *

Deviation from additivity: * *p* < 0.05 (one-tailed Student’s “*t*” test).

## Data Availability

The original contributions presented in this study are included in the article/Supplementary Material. Further inquiries can be directed to the corresponding author.

## Supplementary Materials

The following supporting information can be downloaded at: https://www.mdpi.com/article/10.3390/ijms262211011/s1.

## Abbreviations

The following abbreviations are used in this manuscript:

ABTS^•^	2,2′-azino-bis(3-ethylbenzothiazoline-6-sulfonate) radical
AC	anthocyanins
FRAP	Ferric Reducing Antioxidant Power
GSH	glutathione
IIC	Integrated Interaction Coefficient
SIC	Sample Interaction Coefficient
TEMPOL	4-hydroxy-2,2,6,6-tetramethylpiperidin-1-oxyl
TPTZ	2,4,6-tri(2-pyridyl)-s-triazine
