# Supplementary material for: Interaction of Red Cabbage Extract with Exogenous Antioxidants
Source: Int J Mol Sci. 2025 Nov 14;26(22):11011. doi: 10.3390/ijms262211011 (PMC12652072; doi:10.3390/ijms262211011)
Supplement: Supplementary file 1 [file ijms-26-11011-s001.zip › Supplementary Figure S1.pdf]

Interaction of Red Cabbage Extract with Exogenous Antioxidants

Kacper Kut, Oskar Sitarz, Grzegorz Bartosz, Izabela Sadowska-Bartosz

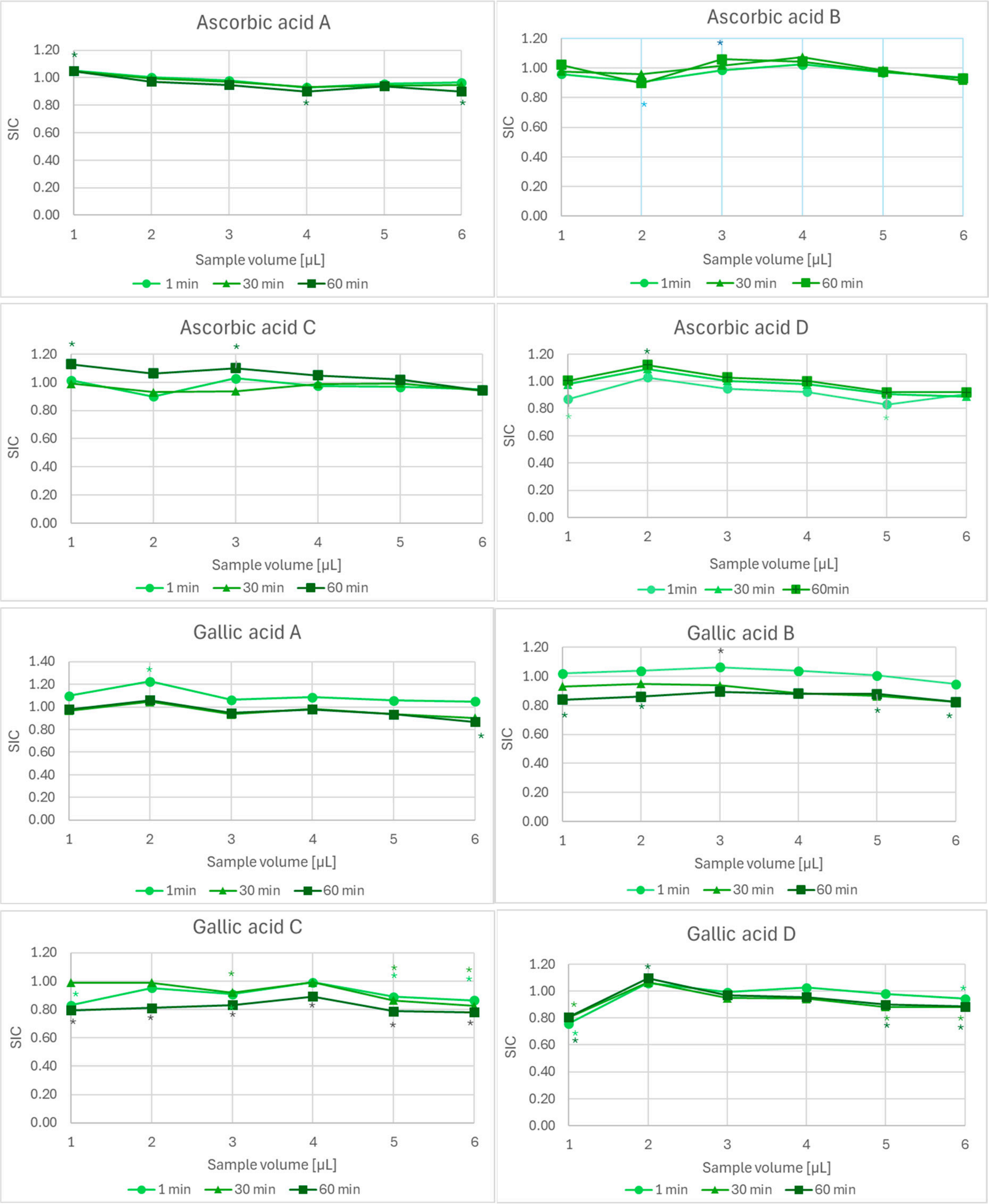

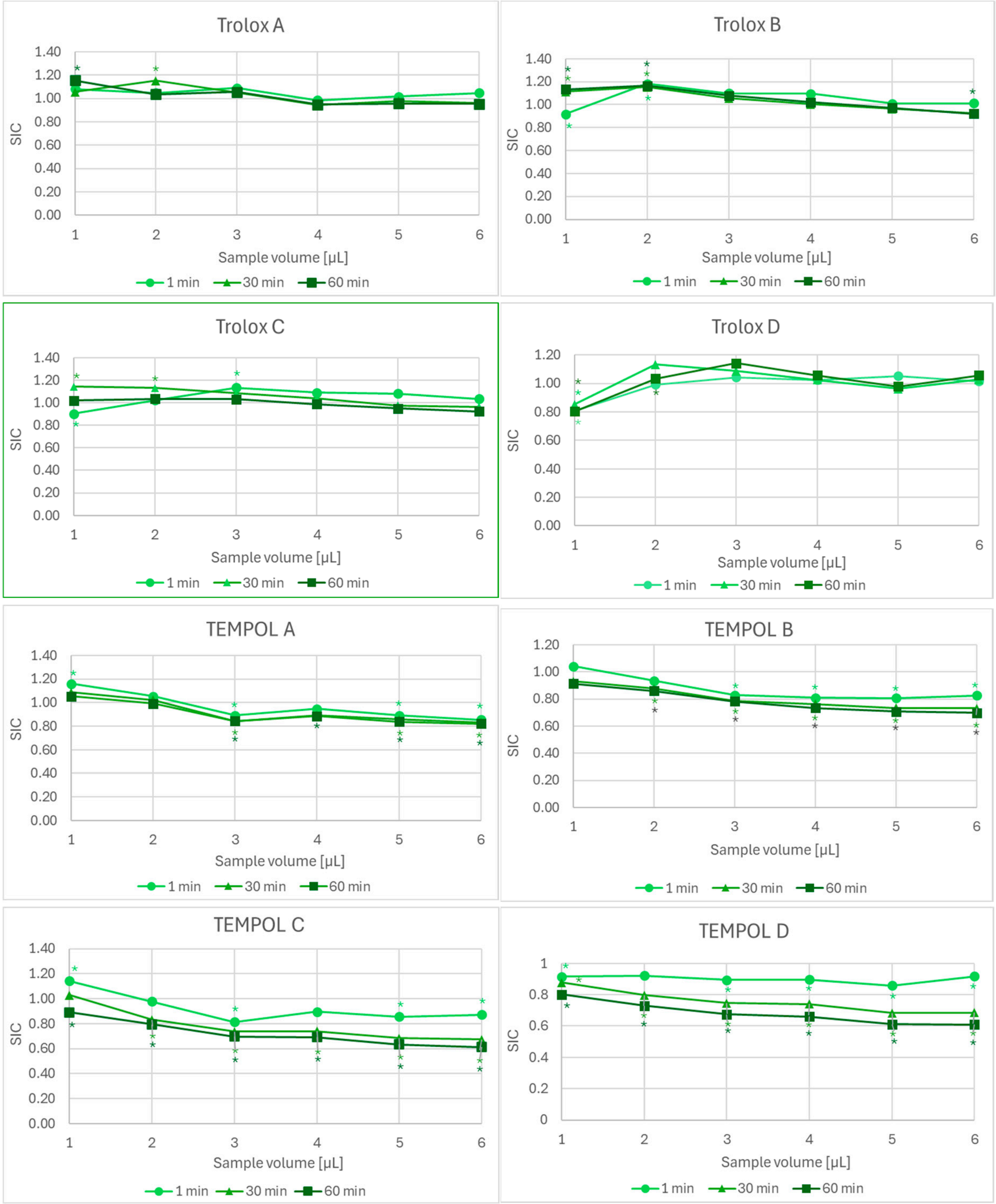

Figure S1. Dependence of the Sample Interaction Coefficient (SIC) on the volume of introduced antioxidant solutions in the ABTS• decolorization assay.
