# Supplementary material for: Interaction of Red Cabbage Extract with Exogenous Antioxidants
Source: Int J Mol Sci. 2025 Nov 14;26(22):11011. doi: 10.3390/ijms262211011 (PMC12652072; doi:10.3390/ijms262211011)
Supplement: Supplementary file 1 [file ijms-26-11011-s001.zip › Supplementary Figure S2.pdf]

# Interaction of Red Cabbage Extract with Exogenous Antioxidants

**Kacper Kut, Oskar Sitarz, Grzegorz Bartosz, Izabela Sadowska-Bartosz**

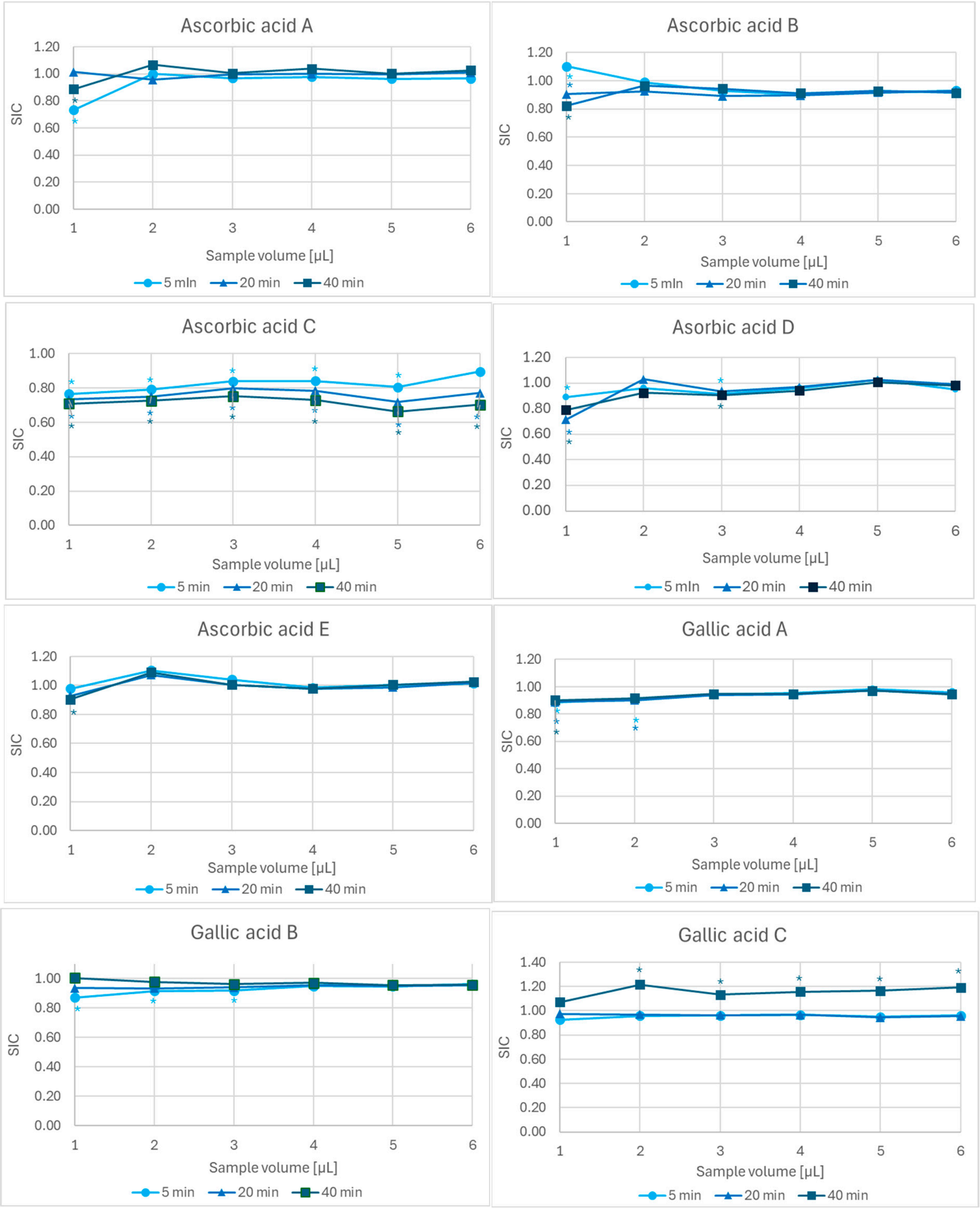

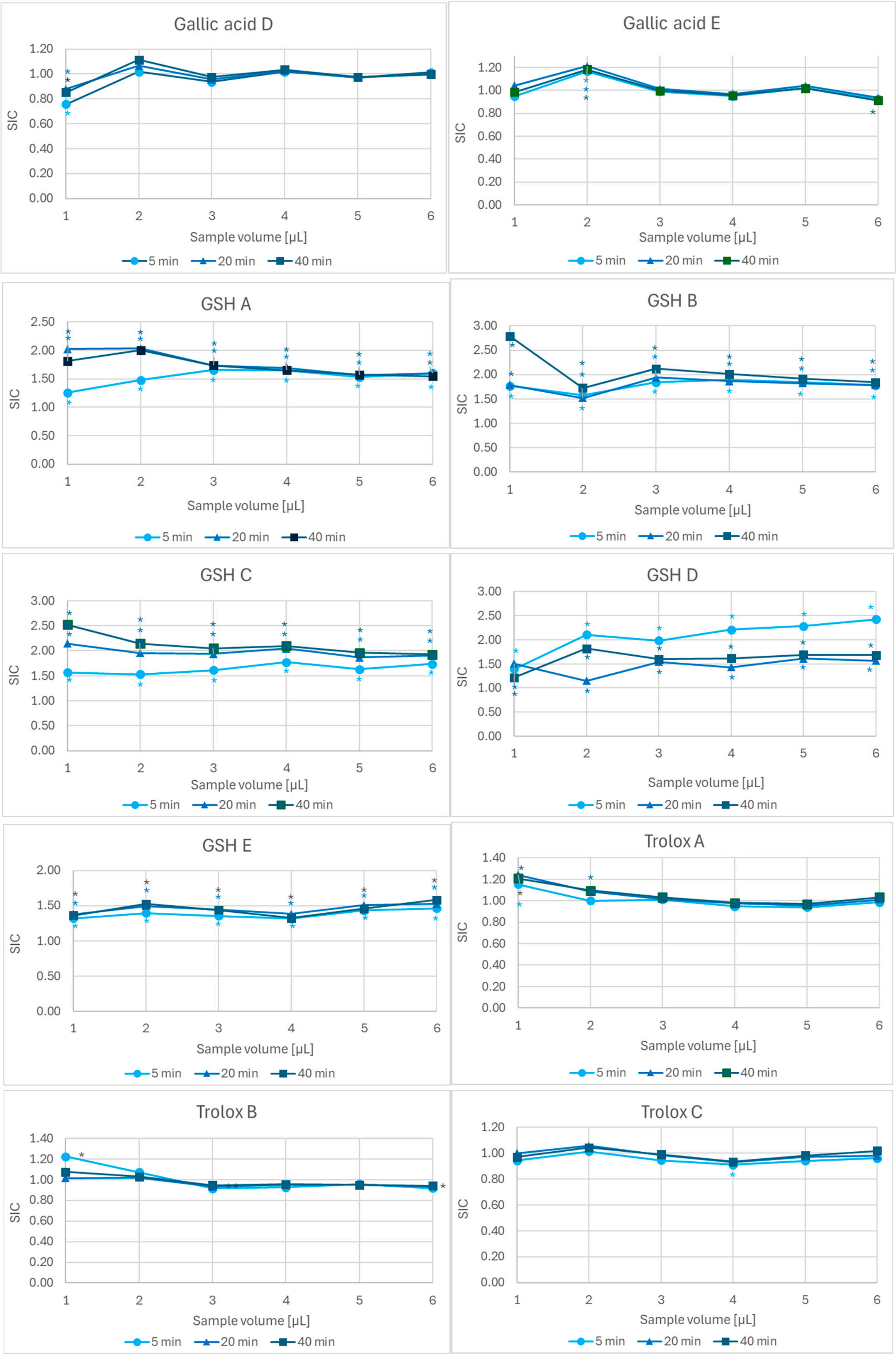
Gallic acid D
SIC
Sample volume [μL]
5 min
20 min
40 min
Gallic acid E
SIC
Sample volume [μL]
5 min
20 min
40 min
GSH A
SIC
Sample volume [μL]
5 min
20 min
40 min
GSH B
SIC
Sample volume [μL]
5 min
20 min
40 min
GSH C
SIC
Sample volume [μL]
5 min
20 min
40 min
GSH D
SIC
Sample volume [μL]
5 min
20 min
40 min
GSH E
SIC
Sample volume [μL]
5 min
20 min
40 min
Trolox A
SIC
Sample volume [μL]
5 min
20 min
40 min
Trolox B
SIC
Sample volume [μL]
5 min
20 min
40 min
Trolox C
SIC
Sample volume [μL]
5 min
20 min
40 min

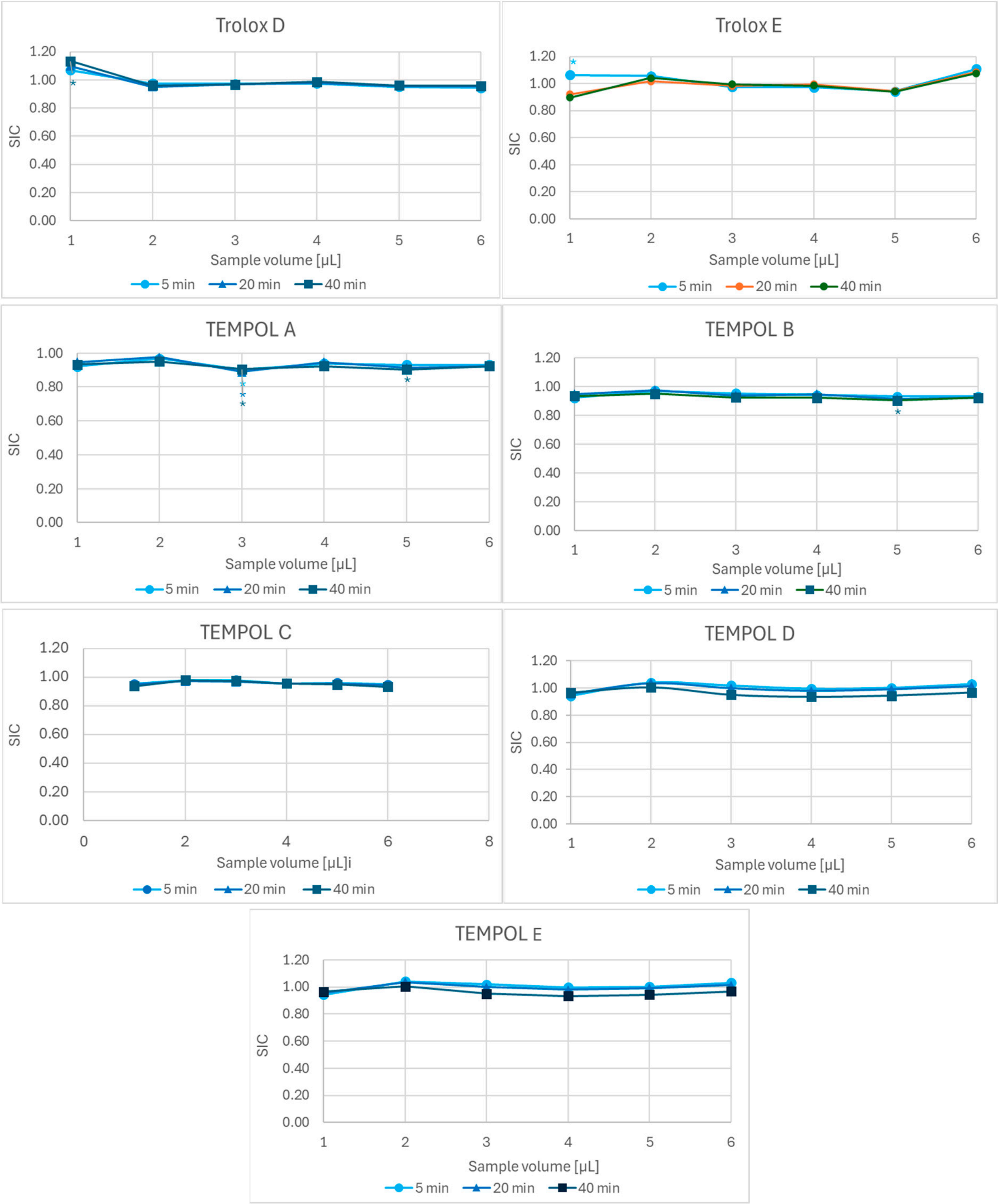


**Figure S2.** Dependence of SIC on the volume of introduced antioxidant solutions in the FRAP assay.
