# Supplementary material for: Interaction of Red Cabbage Extract with Exogenous Antioxidants
Source: Int J Mol Sci. 2025 Nov 14;26(22):11011. doi: 10.3390/ijms262211011 (PMC12652072; doi:10.3390/ijms262211011)
Supplement: Supplementary file 1 [file ijms-26-11011-s001.zip › Supplementary Table S1.pdf]

Interaction of Red Cabbage Extract with Exogenous Antioxidants

Kacper Kut, Oskar Sitarz, Grzegorz Bartosz, Izabela Sadowska-Bartosz

Table S1. Effect of reaction time and extract/antioxidant ratio on the ratio of reaction rates of extract and antioxidants in various reaction times in the ABTS decolorization assay (mean ± SD).

	1 min	30 min	60 min
Ascorbic acid			
A	0.184 ± 0.008	0.263 ± 0.015	0.298 ± 0.012
B	0.577 ± 0.031	0.849 ± 0.051	0.823 ± 0.035
C	1.109 ± 0.049	1.655 ± 0.065	1.756 ± 0.073
D	2.592 ± 0.098	3.731 ± 0.162	3.820 ± 0.117
Gallic acid			
A	0.115 ± 0.043	0.178 ± 0.029	0.201 ± 0.052
B	0.442 ± 0.039	0.492 ± 0.054	0.559 ± 0.068
C	0.819 ± 0.062	1.117 ± 0.071	1.090 ± 0.084
D	2.857 ± 0.126	2.713 ± 0.085	2.864 ± 0.115
GSH			
A	0.270 ± 0.016	0.311 ± 0.009	0.309 ± 0.012
B	0.501 ± 0.028	0.551 ± 0.017	0.575 ± 0.039
C	1.558 ± 0.078	1.344 ± 0.049	1.438 ± 0.091
D	3.403 ± 0.137	3.034 ± 0.096	3.368 ± 0.179
Trolox			
A	0.144 ± 0.025	0.298 ± 0.017	0.277 ± 0.038
B	0.477 ± 0.036	0.797 ± 0.029	0.942 ± 0.056
C	1.133 ± 0.067	1.867 ± 0.101	2.144 ± 0.135
D	3.269 ± 0.149	4.555 ± 0.192	5.240 ± 0.231
TEMPOL			
A	0.614 ± 0.049	0.300 ± 0.023	0.304 ± 0.018
B	1.455 ± 0.097	0.626 ± 0.054	0.619 ± 0.048
C	2.378 ± 0.163	0.830 ± 0.082	0.821 ± 0.064
D	5.251 ± 0.239	1.489 ± 0.088	1.202 ± 0.056
