# Supplementary material for: Interaction of Red Cabbage Extract with Exogenous Antioxidants
Source: Int J Mol Sci. 2025 Nov 14;26(22):11011. doi: 10.3390/ijms262211011 (PMC12652072; doi:10.3390/ijms262211011)
Supplement: Supplementary file 1 [file ijms-26-11011-s001.zip › Supplementary Table S2.pdf]

Interaction of Red Cabbage Extract with Exogenous Antioxidants

Kacper Kut, Oskar Sitarz, Grzegorz Bartosz, Izabela Sadowska-Bartosz

Table 2. Effect of reaction time and extract/antioxidant ratio on the ratio of reaction rates of extract and antioxidants in various reaction times in the FRAP assay.

	5 min	20 min	40 min
Ascorbic acid			
A	1.021 ± 0.016^{Aa}	1.049 ± 0.033^{Ac}	1.067 ± 0.022^{Be}
B	0.904 ± 0.024Cb	0.921 ± 0.045Cd	0.923 ± 0.033Cf
C	0.954 ± 0.013Db	0.970 ± 0.029Dd	0.976 ± 0.038Df
D	0.982 ± 0.011Eb	1.022 ± 0.059Ecd	1.006 ± 0.064Eef
E	0.954 ± 0.047Fab	0.950 ± 0.061Fcd	0.983 ± 0.048Fef
Gallic acid			
A	0.954 ± 0.029	0.897 ± 0.034	0.868 ± 0.016
B	0.964 ± 0.014	0.960 ± 0.052	0.954 ± 0.022
C	0.942 ± 0.043	0.929 ± 0.028	0.912 ± 0.061
D	0.972 ± 0.025	0.980 ± 0.042	0.968 ± 0.015
E	0.965 ± 0.073	0.951 ± 0.032	0.943 ± 0.019
GSH			
A	1.460 ± 0.074	0.720 ± 0.055	0.509 ± 0.038
B	3.426 ± 0.128	1.698 ± 0.077	1.069 ± 0.084
C	6.747 ± 0.271	3.447 ± 0.183	2.202 ± 0.112
D	11.263 ± 0.462	4.014 ± 0.348	3.644 ± 0.098
E	51.979 ± 0.725	38.763 ± 0.674	23.161 ± 0.439
Trolox			
A	0.398 ± 0.013	0.574 ± 0.020	0.636 ± 0.031
B	0.607 ± 0.027	0.877 ± 0.046	0.989 ± 0.038
C	1.433 ± 0.098	1.864 ± 0.074	2.078 ± 0.112
D	2.958 ± 0.123	3.869 ± 0.096	4.423 ± 0.169
E	6.545 ± 0.256	9.663 ± 0.315	11.409 ± 0.384
TEMPOL			
A	0.334 ± 0.014	0.421 ± 0.009	0.400 ± 0.022
B	0.540 ± 0.062	0.648 ± 0.037	0.595 ± 0.012
C	0.821 ± 0.036	0.926 ± 0.054	0.833 ± 0.047
D	1.539 ± 0.082	1.682 ± 0.065	1.544 ± 0.078
E	2.977 ± 0.099	3.070 ± 0.114	2.774 ± 0.081
